# An additional whole-exome sequencing study in 102 panel-undiagnosed patients: A retrospective study in a Chinese craniosynostosis cohort

**DOI:** 10.3389/fgene.2022.967688

**Published:** 2022-09-02

**Authors:** Jieyi Chen, Ping Zhang, Meifang Peng, Bo Liu, Xiao Wang, Siyuan Du, Yao Lu, Xiongzheng Mu, Yulan Lu, Sijia Wang, Yingzhi Wu

**Affiliations:** ^1^ Department of Plastic Surgery, Huashan Hospital, Fudan University, Shanghai, China; ^2^ State Key Laboratory of Genetic Engineering at School of Life Sciences, Fudan University, Shanghai, China; ^3^ CAS Key Laboratory of Computational Biology, Shanghai Institute of Nutrition and Health, University of Chinese Academy of Sciences, Chinese Academy of Sciences, Shanghai, China; ^4^ Center for Molecular Medicine, Pediatrics Research Institute, Children’s Hospital of Fudan University, Shanghai, China; ^5^ The Core Laboratory in Medical Center of Clinical Research, Department of Molecular Diagnostics & Endocrinology, Shanghai Ninth People’s Hospital, State Key Laboratory of Medical Genomics, Shanghai Jiao Tong University School of Medicine, Shanghai, China; ^6^ School of Basic Medical Sciences, Fudan University, Shanghai, China

**Keywords:** craniosynostosis, whole-exome sequencing, genetic diagnosis, research pipeline, candidate variants, cost estimation

## Abstract

Craniosynostosis (CRS) is a disease with prematurely fused cranial sutures. In the last decade, the whole-exome sequencing (WES) was widely used in Caucasian populations. The WES largely contributed in genetic diagnosis and exploration on new genetic mechanisms of CRS. In this study, we enrolled 264 CRS patients in China. After a 17-gene-panel sequencing designed in the previous study, 139 patients were identified with pathogenic/likely pathogenic (P/LP) variants according to the ACMG guideline as positive genetic diagnosis. WES was then performed on 102 patients with negative genetic diagnosis by panel. Ten P/LP variants were additionally identified in ten patients, increasing the genetic diagnostic yield by 3.8% (10/264). The novel variants in *ANKH*, *H1-4*, *EIF5A*, *SOX6*, and *ARID1B* expanded the mutation spectra of CRS. Then we designed a compatible research pipeline (RP) for further exploration. The RP could detect all seven P/LP SNVs and InDels identified above, in addition to 15 candidate variants found in 13 patients with worthy of further study. In sum, the 17-gene panel and WES identified positive genetic diagnosis for 56.4% patients (149/264) in 16 genes. At last, in our estimation, the genetic testing strategy of “Panel-first” saves 24.3% of the cost compared with “WES only”, suggesting the “Panel-first” is an economical strategy.

## Introduction

Craniosynostosis (CRS) is one of the most common congenital craniofacial anomalies in children, with the prevalence of approximately 1/2500 in live births (Lenton et al., 2005). The etiology of CRS is complex. Monogenic, polygenic, chromosomal disorders, and environmental factors might cause CRS in children (Twigg and Wilkie, 2015; Flaherty et al., 2016; Poot, 2019). Previous studies performed different sequencing strategies and focused on genetic diagnosis for CRS patient cohorts (Wilkie et al., 2010; Roscioli et al., 2013; Paumard-Hernandez et al., 2015; Ye et al., 2016; Wilkie et al., 2017; Lee et al., 2018; Topa et al., 2020; Yoon et al., 2020; Tonne et al., 2021; Wu et al., 2021). With the development of the next-generation sequencing (NGS), specific sequencing panels were designed for CRS genetic diagnosis. These panels could achieve 28%–52% diagnostic yield for CRS patients, but the good performance was limited in core CRS genes, such as *FGFR2, FGFR3, ERF, TWIST1, TCF12*, and *EFBN1* (Wilkie et al., 2010; Roscioli et al., 2013; Paumard-Hernandez et al., 2015; Wilkie et al., 2017; Lee et al., 2018; Yoon et al., 2020; Wu et al., 2021). The diagnosis is still complex and ambiguous for a minority of cases. The whole-exome sequencing (WES) is a more general strategy for all exonic genome regions based on NGS. With increasing application of WES in clinical diagnosis and research, many panel-undiagnosed CRS cases have reached positive genetic diagnoses (Miller et al., 2017; Wilkie et al., 2017). Over the last decades, several genes were newly found by WES studies as causal or relevant factors for CRS, like *MEGF8*, *CDC45*, *SMAD6*, *BCL11B*, *TFAP2B*, *SOX6*, and *GINS2* (Twigg et al., 2012; Fenwick et al., 2016; Timberlake et al., 2016; Goos et al., 2019; Timberlake et al., 2019; Tolchin et al., 2020; Nabais Sa et al., 2021). The phenotype of CRS is deeply known as an incompletely penetrant phenotype. CRS is also occasional in many syndromes, which could expand the phenotype spectrums of these syndromes. Therefore, WES is recommended for CRS patients who are difficult to be diagnosed by core genes. Recently, we performed the first genetic research in the Chinese CRS cohort, which sequenced 17 genes known to be associated with CRS, including *FGFR2, FGFR3, TWIST1, EFNB1, TCF12, SKI, RAB23, FGFR1, TGFBR2, POR, SMAD3, ERF, TGFBR1, MSX2, RECQL4, TGFB2, IFT43* (Wu et al., 2021). About half of these patients were identified with pathogenic/likely pathogenic (P/LP) variants, including single nucleotide variants (SNVs) and small insertions and deletions (InDels). However, the etiology of the rest patients in the previous study was unknown. Thus, WES is necessary to be applied to achieve more genetic diagnoses in Chinese patients. On the other hand, the efficiency and cost of different genetic testing strategies for CRS, such as Sanger sequencing, targeted gene panel sequencing and WES, were still understudied.

**FIGURE 1:**
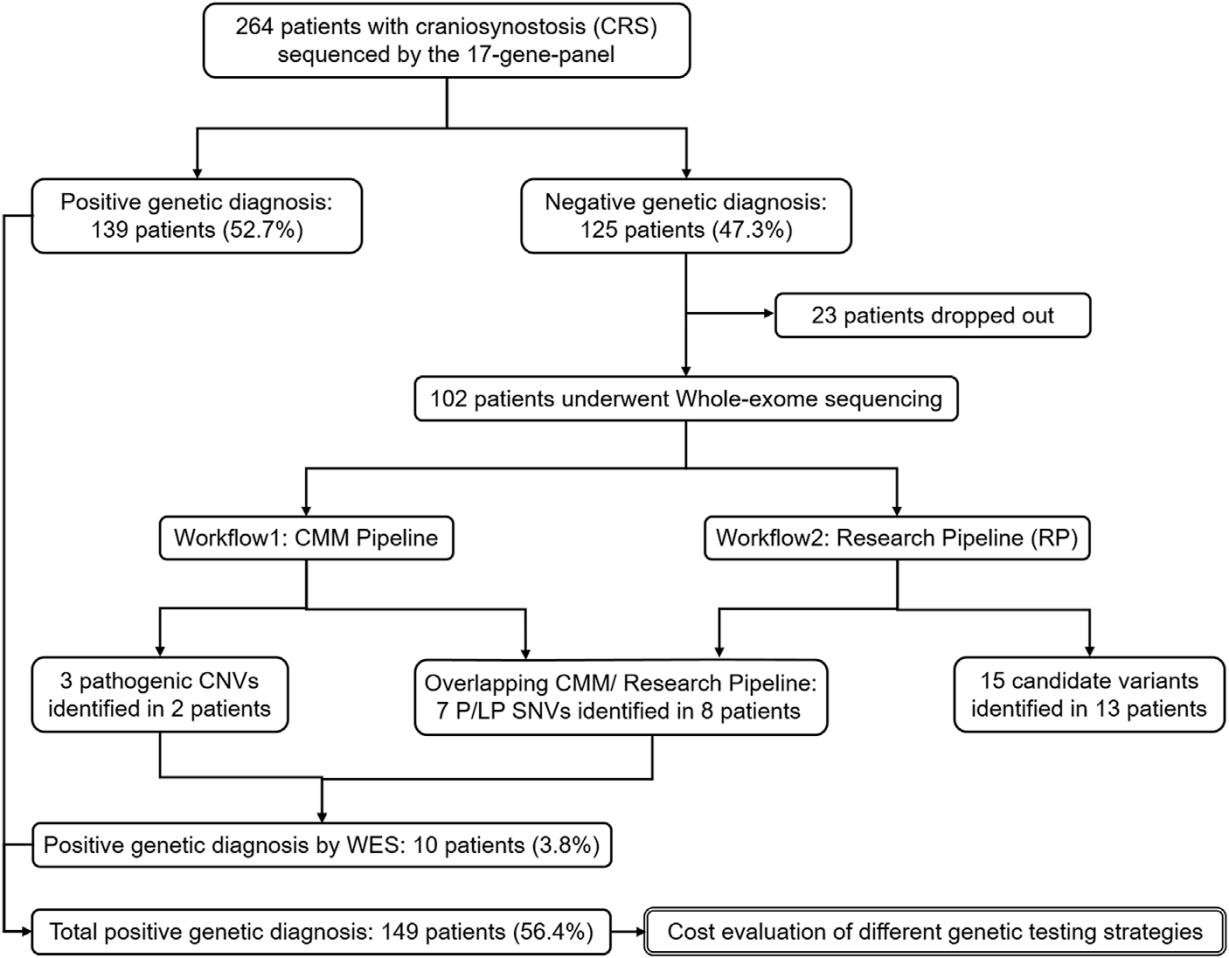
**The framework of the whole study.** Analyses on the genetic diagnosis and variants identification are in single solid line box. Analysis on the economic cost is in double solid line box.

This study was performed following the framework described in Figure 1, as an additional study to 17-gene panel sequencing. We first sequenced 102 panel-negative CRS patients by WES, and then identified variants following two different filter pipelines. Both P/LP and candidate variants on candidate genes of CRS were reported in this study. Subsequently, we summarized all P/LP variants detected by panel and WES as the total genetic diagnosis of the Chinese craniosynostosis cohort. Finally, based on the total genetic diagnosis, we retrospectively estimated the cost of two genetic testing strategies, “Panel-first” and “WES-only”.

## 2 Materials and methods

### 2.1 Patient cohort and sample collection

Patients with premature fusion of one or more cranial sutures indicated by either X-ray or computed tomography and without aplasia/hypoplasia of the cerebrum were diagnosed as CRS. CRS could occur in isolation as non-syndromic CRS (nCRS) or be associated with other clinical manifestations as syndromic CRS (sCRS). The nCRS was defined as cranial vault features occur as an isolated defect. The sCRS was defined as patients with accompanying clinical features in addition to craniofacial deformities, such as digital anomalies, cardiac anomalies, and bone defects. CRS patients visiting or being referred to Huashan Hospital Fudan University during 2017–2021 were recruited. We initially enrolled 264 patients and their available parents into the study, including the 201 patients enrolled in the previous study (Wu et al., 2021) (Supplementary Figure S1). Doctors collected their clinical information and a mouth swab sample for genetic analysis for all the enrolled subjects. The parents completed the checklists of clinical features, and then the doctors confirmed them. All samples in this study were collected with appropriate informed consent and approval of the ethics committee of Huashan Hospital Fudan University (HIRB-2018–007).

### 2.2 DNA extraction and sequencing

DNA extraction and 17-gene-panel sequencing were the same as the previous study (Wu et al., 2021). For WES, the sequencing library was constructed and subjected to the exome sequence capture using an AIExome V1-CNV kit (iGeneTech, Beijing, China). The kit consisted of 26,022 genes in 62 Mb of the target region with mean coverage rate of 99.77% and mean depth of 129. The library was then sequenced on an Illumina platform (Illumina, San Diego, CA, United States) to generate 150-bp paired-end reads.

### 2.3 Data pre-processing for the discovery of variants

We primarily processed the sequencing data by the following steps: 1) reads trimming by Trimmomatic-0.38; 2) sequence alignment to the GRCh37/hg19 human reference genome by Burrows–Wheeler Aligner-0.7.15; 3) sorting and indexing the alignment into BAM format by SAMTools-1.9; 4) marking and removing PCR duplicates by Picard in Genome Analysis Toolkit (GATK-4.1.8).

### 2.4 Variant calling

The variant calling pipeline for panel was described in the previous study, and that for WES was as following.

For SNVs and InDels, best practice recommended by GATK, including BQSR and VQSR, was employed by GATK-4.1.8. In order to reduce false positive rate, we applied an additional quality control to VCF by removing variants with depth <10. Then we re-calibrated the genotype of the remaining variants by the depth ratio of alternative allele. When the depth ratio of alternative allele (DPRA) was less than 0.3, the variant would be defined as “0/0”, 0.3 ≤ DPRA ≤0.7 as “0/1”, and more than 0.7 as “1/1”. We set this threshold according to our experience of Sanger validation.

For copy number variant (CNV) detection, CANOES and HMZDelFinder were separately applied to BAM files and then merged as the previous work (Qin et al., 2018).

### 2.5 Diagnosis pipeline

CMM is short for Center for Molecular Medicine of Children’s Hospital of Fudan University, so the diagnosis pipeline is also named as “CMM pipeline” in this study (Yang et al., 2019). The pipeline included annotation, automatic filtrations, and manual mutation curation according to the American College of Medical Genetics and Genomics (ACMG) interpretation guideline. ACMG interpretation guideline could classify variants into five types, including pathogenic (P), likely pathogenic (LP), uncertain significance (VUS), likely benign (LB) and benign (B) (South et al., 2013). All SNVs, InDels and CNVs were processed in the CMM pipeline.

### 2.6 Research pipeline

We established a research pipeline (RP) focusing on digging candidate variants in all genes which were potentially associated with CRS in previous studies or databases. RP consists of two parts, and the results are merged at the end (Supplementary Figure S2). Only SNVs and InDels were involved in the RP.

In part one, gene-based and filter-based annotation of variants was performed using ANNOVAR. Loss-of-Function (LoF) variants (including frameshift, nonsense, splicing variants) and deleterious missense variants (deleterious score ≥4 or CADD ≥20) were retained. The deleterious score (DS) was referred to the previous study (Calpena et al., 2020). The allele frequency filter was separately filtered with different inheritance models according to gnomAD (v2.1) and Huabiao Project (Hao et al., 2021). When using database of gnomAD, we especially adjusted the threshold of the autosomal dominant model (AD) to 0.00005, considering the allele count ≤20 in 141,456 samples of gnomAD and the estimation method used by Calpena et al. (2020). When using allele frequency from Huabiao Project, we adjusted the threshold of AD to 0.0005, considering the allele count ≤5 in nearly 5000 samples. It is worth mentioning that the inheritance model in 21 only-probands were assumed due to lack of sequencing result from parents.

In part two, Exomiser (Robinson et al., 2014) utilized by “hiPhive” model with “mouse” & “zebrafish”, with the same allele frequency threshold as in part one. We kept only the top 20 variants referring to previous study (Stark et al., 2017).

In the end, the variants both identified in two parts were retained. Then we selected variants meeting with the candidate gene list for CRS (Supplementary Table S1). We made a literature review for genes associated with or causal to CRS. In order to avoid missing, the candidate gene list also consisted of the following parts: gene associated with “Craniosynostosis HP:0001363” or “Abnormal skull morphology HP:0000929” in the database of HPO (Kohler et al., 2021), and all genes of “Craniosynostosis” and “Skeletal dysplasia” in PanelAPP. There were totally 2,259 genes in the list, including 17 genes involved in the previous study (Wu et al., 2021). Finally, we kept the variants whose inheritance model met with that of the gene in OMIM (Amberger et al., 2019).

### 2.7 Validation and confirmation for variants

The candidate variants identified by both the CMM pipeline and RP were validated by Sanger sequencing or qPCR in individuals with sufficient DNA content. Other remained variants were confirmed by the Integrative Genomics Viewer (IGV-2.4.13), because the amount of DNA extracted from mouth swab sample was limited. The result of Sanger sequencing and IGV were extracted in Supplementary Figure S3 and Supplementary Figure S4.

### 2.8 Statistical analysis

We performed contingency table analysis and two-tailed Fisher’s exact tests to assess detailed differences between two or more groups. Single-tailed tests were declared. All statistical analysis was done with R (V4.0.4). *p* ≤ 0.05 was considered as significant.

## 3 Results

### 3.1 Description of patients

In total, 264 patients clinically diagnosed with CRS were continually recruited (Table 1). The sCRS patients account for 61.7% in the whole CRS cohort, and the nCRS patients account for 38.3%. The uni/bi-coronal and multiple synostosis were the main types in sCRS patients. Differently, the sagittal and uni/bi-coronal synostosis were the main types in nCRS patients.

**TABLE 1:** Summary of the CRS patients enrolled.

	Syndromic	Non-syndromic	Combined
	Total	Total	Total
Gender			
Male	87	58	145
Female	76	43	119
Suture fusions			
Sagittal	16	41	57
Metopic	4	1	5
Uni/Bi-coronal	53	36	89
Uni/Bi-lambdoidal	5	10	15
Multiple	54	13	67
Uncertain*	31	0	31
Total	163	101	264

In the panel stage, 59 distinct P/LP variants were identified in 139 patients as positive genetic diagnoses (Supplementary Table S2). The diagnostic yield of the 17-gene panel was 52.7% (139/264), updating that in the previous study. The 102 patients with negative genetic diagnoses by the panel and informed consent were further sequenced by WES in this study, including 21 only-probands and 81 trio-families (Figure 1 and Supplementary Table S3). With over half of sCRS and only a few nCRS patients solved by the panel, 33 sCRS and 69 nCRS patients were left and involved in the WES stage, including 61 males and 41 females.

### 3.2 P/LP variants identified in the WES stage

Following the CMM pipeline, we identified 10 P/LP variants in 10 patients as positive diagnosis, including four SNVs, three InDels, and three CNVs (Table 2). Four variants were in the CRS-related genes, including *ZIC1, SOX6, NFIA, and ARID1B* (Twigg et al., 2015; Bayat et al., 2017; Tolchin et al., 2020; Tonne et al., 2021). The other patients with variants in *ANKH*, *H1-4*, and *EIF5A* had partially consistent phenotypes related to corresponding genes or syndromes according to OMIM (Amberger et al., 2019). For example, case W007 was with laryngomalacia and developmental delay meeting with Rahman Syndrome caused by loss-of-function variants in *H1-4* (Tatton-Brown et al., 2017). Although these three genes were reported to be related to CRS for the first time, they were already known to be related to skeletal dysplasia and skull abnormalities (Kohler et al., 2021). As WES complemented the limitation of CNV detection by the 17-gene panel, we additionally identified the CNV deletions related to the hot CRS gene *TWIST1*, meeting with the well-known haploinsufficiency.

**TABLE 2:** Pathogenic/likely pathogenic (P/LP) Variants identified by the CMM pipeline.

	ID	Sex	Original diagno	Affected suture	Genomic change (GRCh37/hg19)	Gene	Exon	cDNA change	Protein changes	Transcription	Functional change	Inheritance	Classification
SNVs andInDels CNVs	W003	Female	sis syndromic	Bicoronal, Sagittal	chr5:g.14716825delGAA	*ANKH*	exon9	c.1129_1132delinsC	p.F377del	NM_054027	inframe deletion	uncertain	LP
	W007	Female	syndromic	Unilambdoidal	chr6:g.26157052delC	*H1-4*	exon1	c.433_434insC	p.T146Hfs*50	NM_005321	frameshift deletion	de novo	LP
	W016	Female	syndromic	Metopic	chr17:g.7214668G>C	*EIF5A*	intron3	c.271-1G>C	-	NM_001970.5	splicing	de novo	LP
	W033	Female	syndromic	Bicoronal	chr3:g.147131159C>T	*ZIC1*	exon3	c.1165C>T	p.Q389X	NM_003412	nonsense	de novo	P
	W072	Male	non-syndromic	Unilambdoidal	chr11:g.16077306G>A	*SOX6*	exon10	c.1243C>T	p.Q415X	NM_033326	nonsense	de novo	P
	W083	Female	non-syndromic	Sagittal, Metopic	chr1:g.61553899C>T	*NFIA*	exon2	c.106C>T	p.R36X	NM_005595	nonsense	de novo	P
	W097	Male	syndromic	Sagittal	chr6:g.157431670_157431676 delCCAGTCA	*ARID1B*	exon7	c.2346_2352del	p.S784Cfs*59	NM_020732	frameshift deletion	de novo	P
CNVs	W026	Female	syndromic	Sagittal, Bicoronal	chr7:g.16572119_19185044	*TWIST1*					CNV Deletion	de novo	P
	W034	Female	syndromic	Bicoronal	chr7:g.16127149_21956512	*TWIST1*					CNV Deletion	de novo	P
	W053	Female	non-syndromic	Sagittal	chr7:g.18498464_19185044	*TWIST1*					CNV Deletion	uncertain	P

### 3.3 Potentially pathogenic variants

In addition to the positive diagnoses mentioned above, four variants are noticed in the CMM pipeline but were not classified as P/LP variants (Supplementary Table S3). We reported them as “potentially pathogenic variants” in this study.

A frameshift variant of *CDC45* (p.V109fs) in case W030 was defined as a negative diagnosis, because the only one LP variant could not fit to the autosomal recessive (AR) inheritance of *CDC45* (Fenwick et al., 2016; Ting et al., 2020). However, many clinical manifestations of W030 matched Meier-gorlin syndrome, a disease caused by *CDC45*.

A *de novo* nonsense variant of *SNRPB* (p.R94X) in case W019 was classified as VUS in the CMM pipeline. The Cerebro-costo-mandibular Syndrome (CCMS) was caused by the mechanism of nonsense-mediated mRNA decay (NMD) of *SNRPB* (Lynch et al., 2014; Bacrot et al., 2015). This nonsense variant was predicted to efficiently trigger NMD by NMDetective-A, with the score of 0.85 (Lindeboom et al., 2019).

W019 was with cor triatrium, atrial septal defect, atresia of the external auditory canal, and self-report short stature for her age, but without other typical manifestations of CCMS, such as micrognathia or rib abnormalities by X-ray examination. Besides, CRS was never reported as a phenotype of CCMS, which decreasing the classification of the variant.

Although several studies have reported that the variants of *IL11RA* could cause Crouzon-like craniosynostosis in AR inheritance (Keupp et al., 2013; Miller et al., 2017; Brischoux-Boucher et al., 2018), the two compound heterozygotes of *IL11RA* (p. [P243R], [L236P]) in case W093 were classified as VUS in the CMM pipeline.

### 3.4 Candidate variants identified by the research pipeline

In addition to genetic diagnosis, we performed the research pipeline (RP) to look for candidate variants according to the candidate gene list for CRS (details in Supplementary Table S3). In total, 62 variants were identified in 42 cases by the automatic part of RP (Supplementary Table S3). In order to identify the candidate variants for CRS, we excluded all LB/B variants according to ACMG criteria manually. As a result, all the nine P/LP SNVs and InDels identified in the CMM pipeline and another 16 variants were remained. After reviewing all the genes of remaining variants, we excluded *APC*, which were mainly associated with cancers. Finally, we listed 15 variants of 11 genes as candidate variants for 13 CRS cases, including the compound heterozygotes of *IL11RA* (Table 3).

**TABLE 3:** Candidate variants identified by the research pipeline (RP).

ID	Sex	Original diagnosis	Suture fusions	Proband HPO records	Genomic change (GRCh37/hg19)	Gene functional change	CADD	DS	Exomiser rank	Inheritance	Genetic model^a^	ACMG classification	Supporting evidences
W001	Male	non-syndromic	Sagittal	HP:0001363,HP:0004442	chr5:g.140966698A>G	*DIAPH1*, NM_005219, exon3, c.T211C, p.S71P	22.9	5	1	uncertain	AD (assumed)	VUS	Ercan-Sencicek et al. (2015)
W008	Male	non-syndromic	Sagittal	HP:0001363,HP:0004442	chrX:g.79988961C>G	*BRWD3*, NM_153252, exon12, c.G1121C, p.G374A	26.7	2	1	maternal (het)	XLR	VUS	Field et al., 2007, Tarpey et al. (2009)
W015	Male	non-syndromic	Sagittal	HP:0001363,HP:0004442	chr4:g.5798945C>A	*EVC*, NM_153717, exon14, c.C2083A, p.L695M	25.7	2	2	uncertain	AD (assumed)	VUS	Fischer et al., 2015, Tiberio et al. (2021)
W021	Male	syndromic	Bicoronal,	HP:0001363,HP:0011324,	chr9:g.109688611C>G	*ZNF462*, NM_021224, exon3, c.C2418G, p.N806K	20.7	1	17	uncertain	AD (assumed)	VUS	Weiss et al., 2017, Kruszka et al. (2019)
			Unilambdoidal	HP:0000750,HP:0001270								
W023	Male	non-syndromic	Unicoronal	HP:0001363,HP:0004440	chr17:g.41833050G>A	*SOST*, NM_025237, exon2, c.C302T, p.T101I	23.5	4	1	uncertain	AD (assumed)	VUS	Kim et al. (2011)
W038	Male	non-syndromic	Sagittal	HP:0001363,HP:0004442	chr4:g.5733339C>T	*EVC*, NM_153717, exon4, c.C572T, p.T191I	27.1	1	3	uncertain	AD (assumed)	VUS	Fischer et al., 2015,
	Tiberio et al. (2021)											
W041	Male	non-syndromic	Bilambdoidal	HP:0001363,HP:0004443	chrX:g.50378636C>A	*SHROOM4*, NM_020717, exon4, c.G437T, p.R146L	33	4	18	maternal (het)	XLR	VUS	Lopes et al., 2016, Amberger et al. (2019)
W043	Male	non-syndromic	Bicoronal, Unilambdoidal	HP:0001363,HP:0011324	chrX:g.63410654A>G	*AMER1*, NM_152424, exon2, c.T2513C, p.L838S	24.2	4	1	maternal (het)	XLR	VUS	Comai et al., 2018, Heikoop et al. (2021)
W051	Male	non-syndromic	Sagittal	HP:0001363,HP:0004442	chrX:g.50345801C>A	*SHROOM4*, NM_020717, exon7, c.G3774T, p.Q1258H	23.2	3	19	maternal (het)	XLR	VUS	Lopes et al., 2016, Amberger et al. (2019)
W071	Male	non-syndromic	Sagittal	HP:0001363,HP:0004442	chrX:g.148037734C>T	*AFF2*, NM_002025, exon11, c.C2159T, p.S720F	29.1	5	8	maternal (het)	XLR	VUS	Knight et al. (1996)
W085	Male	non-syndromic	Sagittal	HP:0001363,HP:0004442	chrX:g.129149626G>A	*BCORL1*, NM_021946, exon3, c.G2878A, p.D960N	21.2	2	7	maternal (het)	XLR	VUS	Shukla et al. (2019)
W093	Female	non-syndromic	Sagittal, Bicoronal	HP:0001363,HP:0011324	chr9:g. [34658598C>G]; [34658577T>C]	*IL11RA*, NM_001142784, exon8, c. [C728G]; [T707C], p. [P243R]; [L236P]	27.6/27.4	5/5	5	paternal/maternal	AR	VUS/VUS	Keupp et al., 2013, Miller et al., 2017, Brischoux-Boucher et al. (2018)
W095	Male	syndromic	Sagittal	HP:0001363,HP:000444, HP:0001627,HP:0000750	chr7:g. [21726797A>C]; [21639655T>C]	*DNAH11*, NM_001277115, exon33; exon15, c. [A5702C]; [T2918C], p. [E1901A]; [V973A]	25.1/24.8	6/3	1	paternal/maternal	AR	VUS/VUS	El Zein et al., 2003, Tiberio et al. (2021)

a AD, autosomal dominant; AR, autosomal recessive, XLR = X-linked recessive.

b All candidate genes mentioned here were associated with at least one of HP:0001363 or HP:0000929, also shown in Supplementary Table S1. Besides, we show other associated HPOs, in this column may be related to CRS.

The recurrence of the same candidate gene in different patients also increased its potential of pathogenicity. Among all pathophysiology of CRS, the genes orchestrating the primary cilium structure and function were also known to play an important role (Tiberio et al., 2021). The *EVC* encodes a positive regulator downstream the HH signaling, expressed on the ciliary membrane as a single-pass transmembrane protein. We found two sagittal patients, case W015 and W038, carried heterozygous missense variants in *EVC*. To date, only a single case of sagittal synostosis has been reported in a patient affected by Ellis-van Creveld syndrome, but this disorder is usually caused by homozygous variants in either the *EVC* or *EVC2* genes (Fischer et al., 2015; Tiberio et al., 2021). Although there was no other candidate variants and inheritance validation composing compound heterozygotes in this study, the candidate variants suggest attention on *EVC*. Another candidate gene identified in more than one patient was *SHROOM4*. Case W041 and W051 carried different maternally inherited X linked missense variants. The Stocco dos Santos type of X-linked syndromic intellectual developmental disorder and Rett-like syndrome are caused by variants in the *SHROOM4*, with microcephaly as an occasional phenotype (Lopes et al., 2016; Amberger et al., 2019). Although the parents of two patients did not report intellectual developmental delay for the moment, we will perform a long-term follow-up in the future.

Among other candidate genes with only one case, they are associated with HPO of skeletal dysplasia (Supplementary Table S1). *ZNF462* and *IL11RA* are directly associated with CRS. Besides, we tried to look out the indirect linkages between candidate genes and CRS. The syndromes or intellectual developmental disorders caused by variants in *DIAPH1*, *AFF2*, *BCORL1* and *BRWD3* are occasionally with abnormalities of cranium, such as microcephaly or macrocephaly tall forehead. Furtherly, *SOST* and *AMER1* were causal for cranial sclerosis with craniofacial abnormalities. *DNAH11* encodes critical protein for cilia, which might impact development of cranial sutures the primary cilium structure and function (Tiberio et al., 2021). The supporting evidences for above were listed in the last column of Table 3. In additional to literature review, we furtherly analyzed the gene expressions in the cranial neural crest cells (CNCCs), one of the critical cell resources for cranial sutures (Flaherty et al., 2016). Based on the published RNAseq data in GSE70751 (Prescott et al., 2015), we found that the expression levels of *SHROOM4*, *BRWD3*, *AMER1*, *EVC*, *AFF2* and *ZNF462* were about or at the top 10% in human CNCCs (Supplementary Figure S5).

### 3.5 Summary of the total genetic diagnosis

**FIGURE 2:**
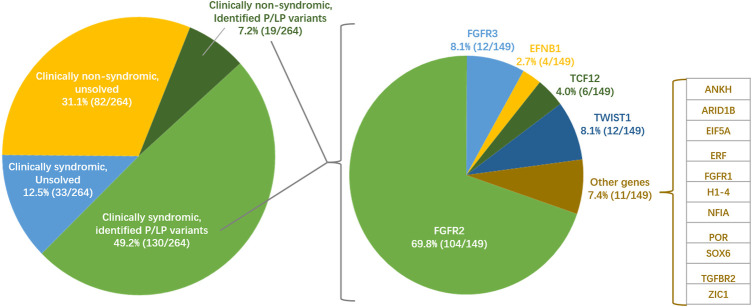
**Situation of clinical diagnosis, genetic diagnosis and pathogenic genes of craniosynostosis in the Chinese cohort of this study (*n* = 264)**. The pie chart on the left shows a broad classification based on clinical diagnosis and genetic diagnosis in this study. The pie chart on the left shows the diagnosis contribution of 16 pathogenic genes in positive genetic diagnosis.

In this study, we only defined patients with P/LP patients as positive genetic diagnosis, but potentially pathogenic and candidate variants as negative. The hot gene exonic capture by 17-gene panel in the first stage and the overall exonic capture by WES in the second stage are subset of NGS. We integrated all positive genetic diagnoses in the two stages as the total genetic diagnostic performance of exonic NGS. We totally identified 69 P/LP variants in 16 genes in this study. *FGFR2* contributed the most diagnostic yield among all genes (Figure 2). *FGFR3*, *TWIST1*, *TCF12*, and *EFNB1* were the following contributor for diagnostic yield. The rest 11 genes only contributed genetic diagnosis for one patient separately.

The overall diagnostic yield was 56.4% (149/264). Significantly more positive diagnoses were found in syndromic (79.8%) than that in non-syndromic (18.8%) patients (one-tailed *p* = 3.78 × 10^–23^, Figure 2). The diagnostic yields were also different among different suture fusion types (*p* = 1.30 × 10^–15^, Supplementary Table S4). To specific suture synostosis, we found that the patients with bi/uni-coronal and multiple synostosis tended to get positive genetic diagnoses than patients with other types (one-tailed, P_Coronal_ = 3.28 × 10^–3^, P_Multiple_ = 3.50 × 10^–6^, Supplementary Table S4).

### 3.6 Cost evaluation of different genetic testing strategies

Our study used a genetic testing strategy of “Panel-first and then WES”, which means that we first performed the 17-gene panel genetic testing for CRS patients, and only for panel-negative patients, we would perform the WES. In order to compare the cost between two strategies, we estimated the cost of two situations based on positive genetic diagnoses integrated above. Since the 17-gene panel included all exons of 17 genes, all P/LP variants identified by the 17-gene panel could also be identified by WES. Thus, the diagnostic yield of the a “WES-only” strategy would be same as the “Panel-first” strategy. However, the costs of these two strategies are different. The average fee of a trio-WES was about $945, and 17 gene-panel for a trio was about $315. We retrospectively estimated the cost in all 239 patients who underwent only panel or both panel and WES (Table 4). At the same diagnostic yield, the genetic testing strategy of “Panel-first” ($715 per trio family) could save 24.3% cost ($230) on genetic testing for probands’ families in average, rather than “WES-only” ($945 per trio family).

**TABLE 4:** Cost estimation for two genetic testing strategies.

Genetic testing strategy	Panel_Pos N = 139 (57.3%)	WES_Pos N = 10 (4.2%)	Both_Neg N = 92 (38.5%)	Total N = 241	Estimated cost for per-trio
Panel-first	$43,785	$12,600	$115,920	$172,305	$715
WES-only	$131,355	$9,450	$86,940	$227,745	$945

Panel_Pos: The cases got positive diagnosis only by the 17-gene panel sequencing.

WES_Pos: The cases got positive diagnosis by WES, but negative by the 17-gene panel sequencing.

Both_Neg: The cases got negative diagnosis by both 17-gene panel sequencing and WES.

## 4 Discussion

### 4.1 Increased diagnostic yield by WES

Among all 264 patients involved in our genetic testing study, the diagnostic yield of the 17-gene panel was 52.7%, and the additional diagnostic yield by WES was 3.8% (10/264, Table 2), which might mainly due to the two reasons in this study. The first one is the additional detection of CNVs. The panel used in the first stage could only detect SNPs and InDels, so the three deletions of *TWIST1* were missed in the panel stage. The other reason is the expand for more gene detection by WES than the 17-gene panel. However, the actual performance of WES might be better than the current observation in this study. The exclusion of 23 patients lost to follow-up and lacking of functional experiments for VUS might decrease the diagnostic yield of WES. Another reason for underestimation of diagnostic yield is the detection of CNVs might be missed. The CNV calling pipeline used in this study depend on at least 20 samples from the same batch (Qin et al., 2018), but we sequenced samples in many different batches in practice. Thus, the sample size of many batches is fewer than ten, resulting failure of CNV detection.

Among all seven P/LP SNVs and InDels, only two of *ZIC1* and *NFIA* were reported, while the other diagnosed variants are novel. They exactly gave the genetic diagnosis to patients’ families and doctors, and expanded the variant spectra of CRS. The phenotype spectra of several gene-related diseases were also expanded. For example, we were not aware of previous descriptions of CRS associated with variants in *ANKH*, *H1-4*, and *EIF5A*, but the clinical features and the functional of these variants identified were considered sufficient to assign positive diagnosis. To the four candidate variants identified in W019, W030 and W093, although they could not lead to positive diagnosis temporarily, they pointed out the direction of experimental verification indicating their physiology for CRS.

### 4.2 Candidate variants identified by different pipelines

Clinical interpretation of genomic variants requires the standard classification guidelines and workflows, as well as considering the consistency between a variant and a disease phenotype by enough solid evidence. Research-based analysis performed by the Clinical Genetics Group, Oxford (CGG) identified additionally over one-fold P/LP variants than the GE/GMC pipeline for rare diseases, which demonstrated the value of research analysis and the importance of continually improving algorithms to maximize the potential of clinical genome sequencing (Hyder et al., 2021). Our study combined the CMM pipeline following the ACMG guideline and the RP as a supplement. These ACMG germline variant curation guidelines have been broadly adopted by clinical genetic testing laboratories globally to report genetic diagnoses of genetic diseases (Niehaus et al., 2019). However, the limitation of clinical practice for genetic diagnosis is the strictness and rigorousness. It is difficult to explore more novel genes for CRS by the CMM pipeline only. Thus, we referred to several studies and established the RP to identify candidate variants for genetic diagnosis to support further research.

All the P/LP variants and one potentially pathogenic variant identified by the CMM pipeline could be covered by the RP. Moreover, the RP could identify more candidate variants classified as VUS by the ACMG guideline. Although these specific RP variants were not validated by experiments in this study, they could serve as evidence to look for any other patients with variants in the same genes or a potential direction of functional experiments. For example, the gene of *EVC* and *SHROOM4* were reported in more than one case among candidate variants identified by RP, which should be considered for functional validation first. The indirect linkages between the rest candidate genes and CRS could be inferred by literature review. The high expression of *SHROOM4*, *BRWD3*, *AMER1*, *EVC*, *AFF2*, and *ZNF462* in CNCCs suggested the probable pathophysiology of CRS from another perspective.

In sum, the CMM pipeline is reliable for genetic diagnosis, while the RP is a loose analysis process to identify both P/LP variants and candidate variants. The shortage of both pipelines is the requirement of manual work in ACMG classification, which is also a worldwide challenge. On the other hand, since the RP is specific to the research for CRS, the current list of candidate genes only considered skeletal abnormalities as the cause of CRS, but ignored other possibilities of CRS as a secondary phenotype of other disorders.

### 4.3 Recommendations of genetic testing for CRS

With the comparison between sCRS and nCRS patients, the NGS based on exonic regions significantly contributed more to the genetic diagnosis in sCRS, regardless of panel or WES in this study. Such difference between sCRS and nCRS was also reported in previous studies (Roscioli et al., 2013; Wilkie et al., 2017; Lee et al., 2018; Yoon et al., 2020). Meanwhile, the significantly higher genetic diagnostic yield was observed in the patients with bi/uni-coronal or multiple synostosis. Therefore, the genetic testing, especially in exonic genome regions, is recommended for sCRS patients and patients with bi/uni-coronal or multiple synostosis for genetic diagnosis.

There are many genetic testing strategies for CRS in previous studies conducted in different countries (Kutkowska-Kazmierczak et al., 2018). In general, the doctor should give a primary clinical diagnosis and recommend a genetic testing, seemed like a “Step-by-step” strategy, including Sanger sequencing of specific exons of *FGFR2*, *FGFR3*, and *TWIST1*, and structural variants detection around *TWIST1*. However, the match of primary clinical diagnosis and proper genetic testing depends on the experience of doctors, so the “Step-by-step” strategy often lasts a long time and cause financial burden, also known as a long diagnosis odyssey. With the increasing knowledge about CRS genes and the advances of NGS, several sequencing panels were designed for CRS (Lee et al., 2018; Yoon et al., 2020; Tonne et al., 2021; Wu et al., 2021). The reported panels could cover all the exons in the “Step-by-step” strategy or core genes for CRS in only one step. Therefore, we proposed “a CRS sequencing panel first and then WES for panel-negative patients” strategy (Panel-first strategy) as a new “Step-by-step” strategy for genetic testing of CRS patients, of which the efficiency is not limited by the experience of doctors.

As WES is recommended as the first-tier for genetic testing in comprehensive hospitals in China (Yang et al., 2013; Hu et al., 2017), we compared the price of “Panel-first” and “WES-only”. We found the cost of former is 24.3% lower compared with the latter in average. Since over 50% patients could get genetic diagnoses only by the 17-gene panel, it is obvious that the “Panel-first” strategy has a lower cost without losing the genetic diagnostic yield, which is more suitable for price-sensitive patients and their families. On the other hand, only several hospitals and doctors are famous for diagnosing and treating CRS, causing resource imbalance. Thus, we especially recommend a “Panel-first” strategy to the hospitals with a large number of CRS patients.

There are three limitations to the estimation in our study. The first one is about the price. We used the present prices in only a few three-A hospitals in Shanghai as reference, regardless of price fluctuation and regional difference. The second is about the gene selection of panel. The total genetic diagnosis covered 16 genes, while only five genes, including *FGFR2*, *FGFR3*, *TWIST1*, *TCF12*, and *EFNB1* contributed most. Thus, “which genes should be selected in the panel with the highest efficiency”, should be considered in the future study. Due to the limited budget and sample size, we only performed the “Panel-first” strategy in the study, and assumed all positive cases could be detected in the group of “WES-only”. Independent groups of different strategies and their real cost should be compared to make more precise estimation. Despite the limitations mentioned above, estimating the cost of different genetic testing strategies could be useful for clinical practice.

## Data Availability

The datasets for this article are not publicly available due to concerns regarding participant/patient anonymity. Requests to access the datasets should be directed to the corresponding author.
