# Supplementary material for: An additional whole-exome sequencing study in 102 panel-undiagnosed patients: A retrospective study in a Chinese craniosynostosis cohort
Source: Front Genet. 2022 Sep 2;13:967688. doi: 10.3389/fgene.2022.967688 (PMC9481236; doi:10.3389/fgene.2022.967688)
Supplement: Supplementary file 1 [file Image1.PDF]

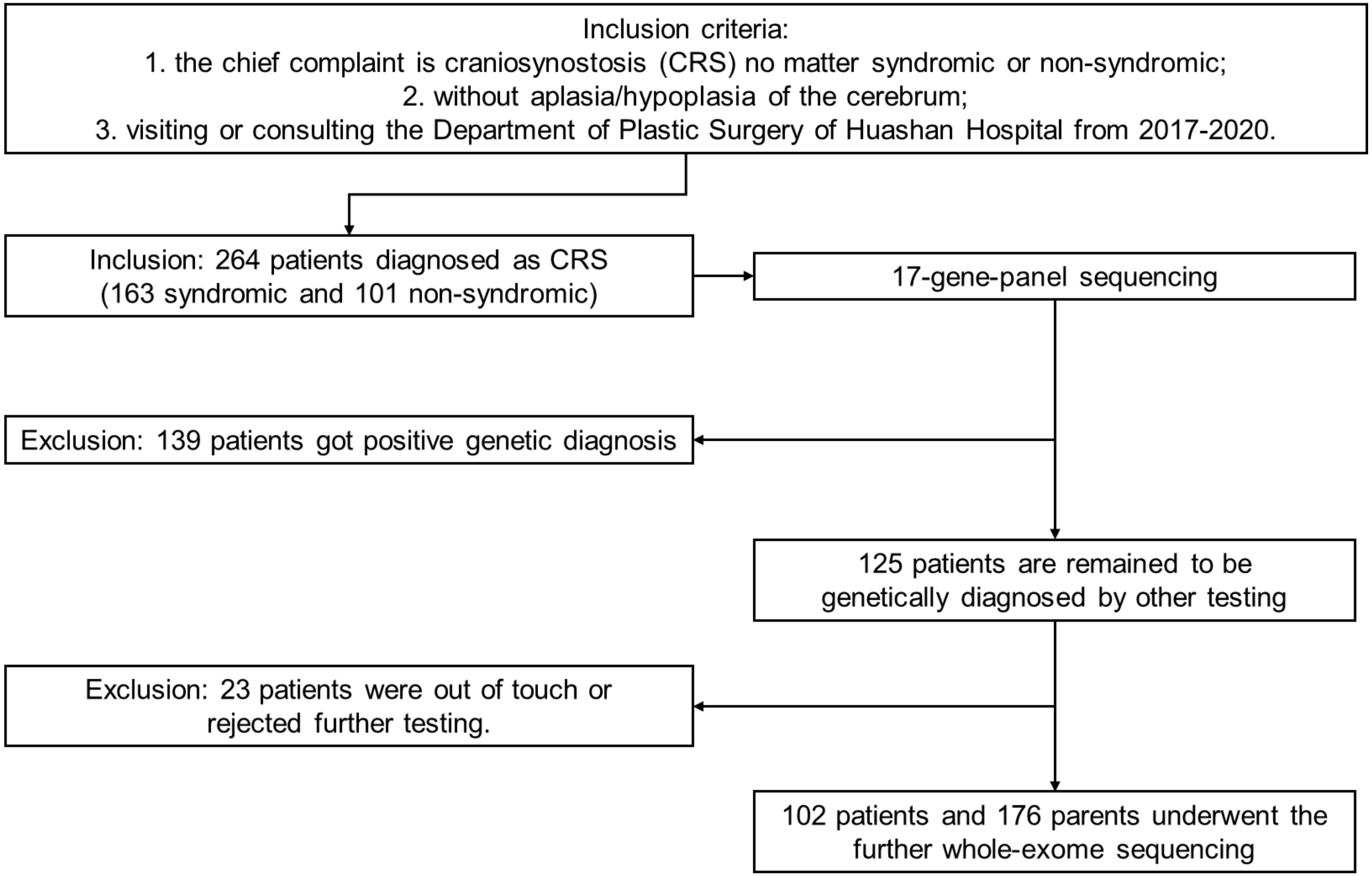

Supplementary Figure 1. The flow chart of sample selection.

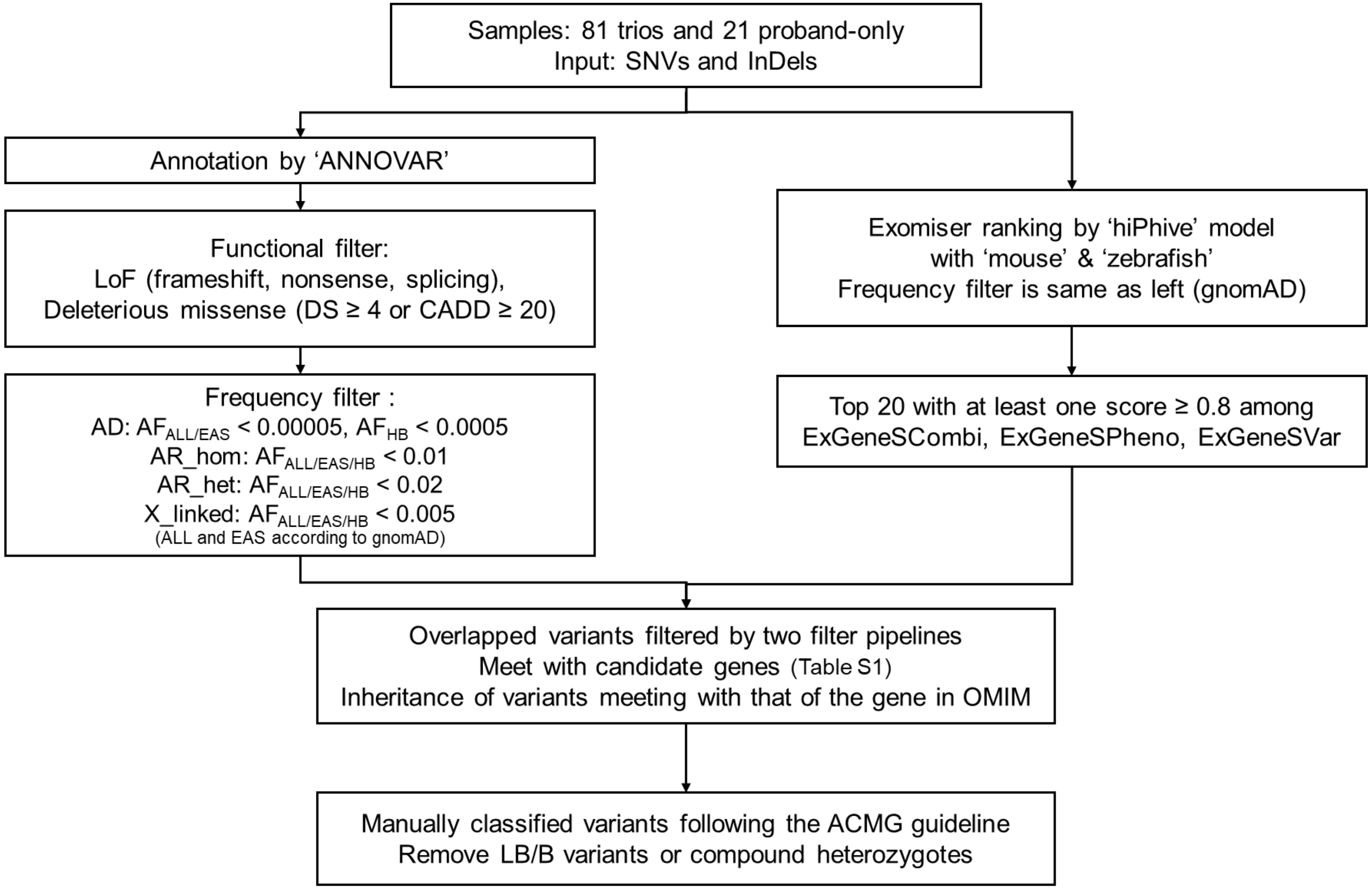

Supplementary Figure 2. The research pipeline to identify potential diagnosis.

Screenshot in the proband	Screenshot in the father	Screenshot in the mother
W003 - chr5:g.14716825delGAA,ANKH,NM_054027,exon9,c.1129_1132delinsC,p.F377del		
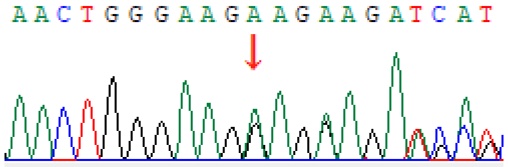	none	none
W007 - chr6:g.26157052delC,H1-4,NM_005321,exon1,c.433_434insC,p.T146Hfs*50		
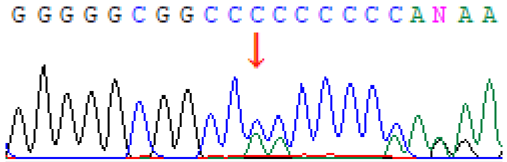	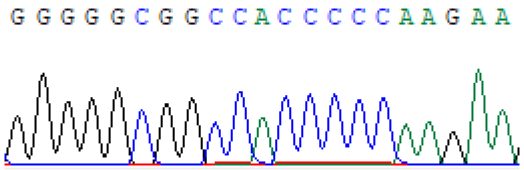	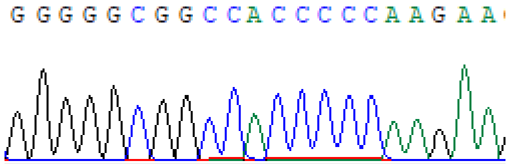
W016 - chr17:g.7214668G>C,EIF5A,NM_001970,exon4,c.271-1G>C,splicing		
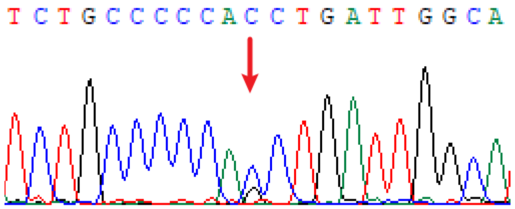	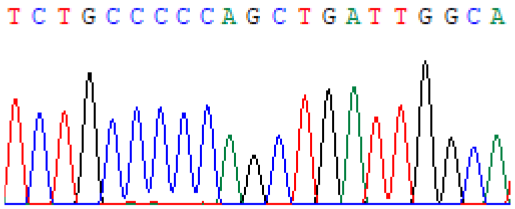	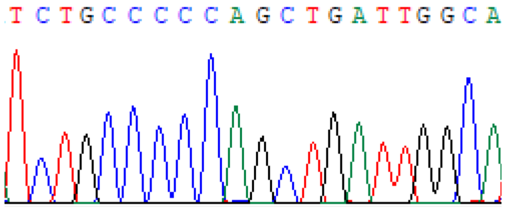

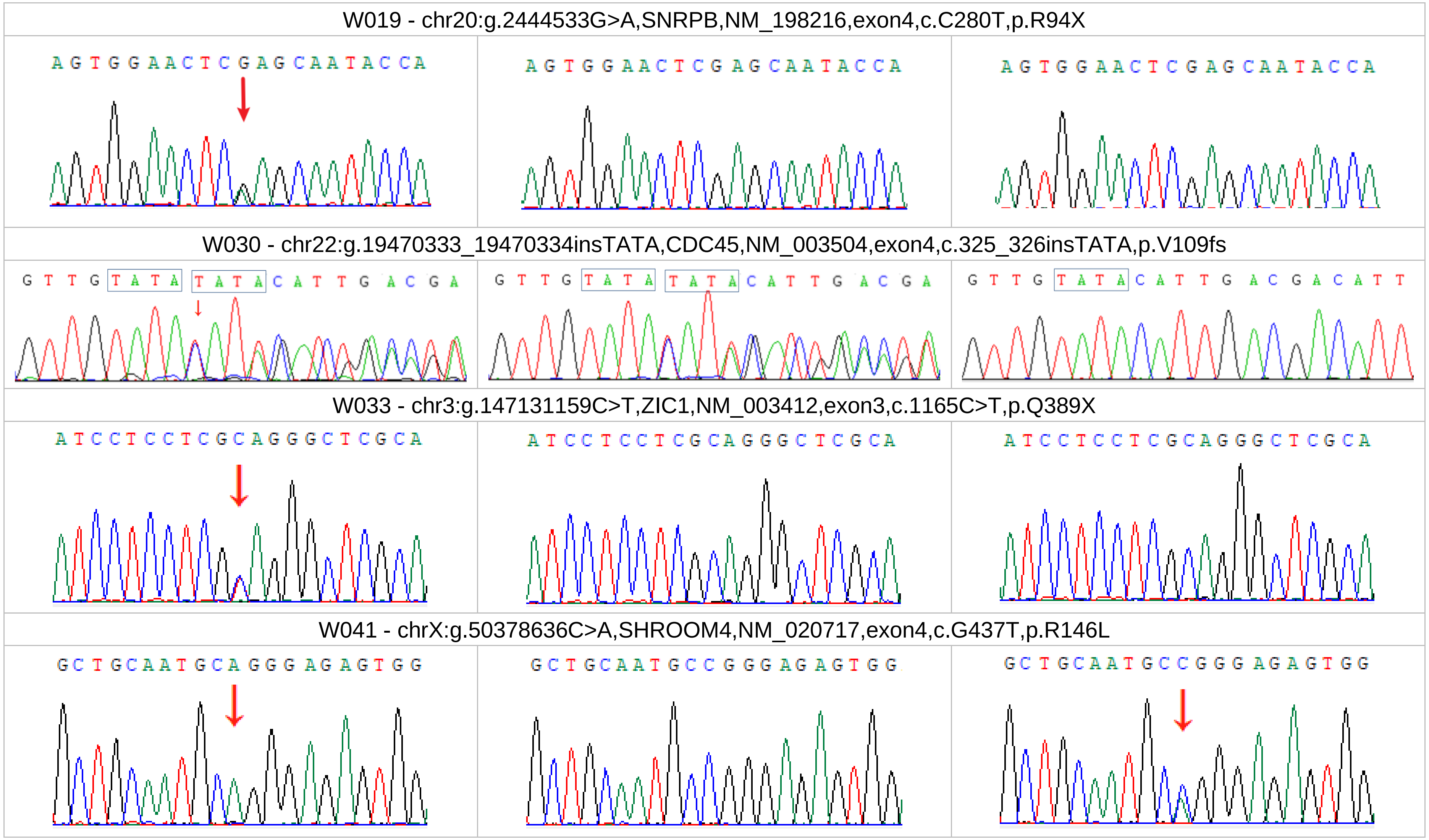
W019 - chr20:g.2444533G>A,SNRPB,NM_198216,exon4,c.C280T,p.R94X
AGTGGAACTCGAGCAATACCA
AGTGGAACTCGAGCAATACCA
AGTGGAACTCGAGCAATACCA
W030 - chr22:g.19470333_19470334insTATA,CDC45,NM_003504,exon4,c.325_326insTATA,p.V109fs
G T T G T A T A T A T A C A T T G A C G A
G T T G T A T A T A T A C A T T G A C G A
G T T G T A T A C A T T G A C G A C A T T
W033 - chr3:g.147131159C>T,ZIC1,NM_003412,exon3,c.1165C>T,p.Q389X
ATCCTCCTCGCAGGGCTCGCA
ATCCTCCTCGCAGGGCTCGCA
ATCCTCCTCGCAGGGCTCGCA
W041 - chrX:g.50378636C>A,SHROOM4,NM_020717,exon4,c.G437T,p.R146L
GCTGCAATGCAGGGAGAGTGG
GCTGCAATGCCGGGAGAGTGG
GCTGCAATGCCGGGAGAGTGG

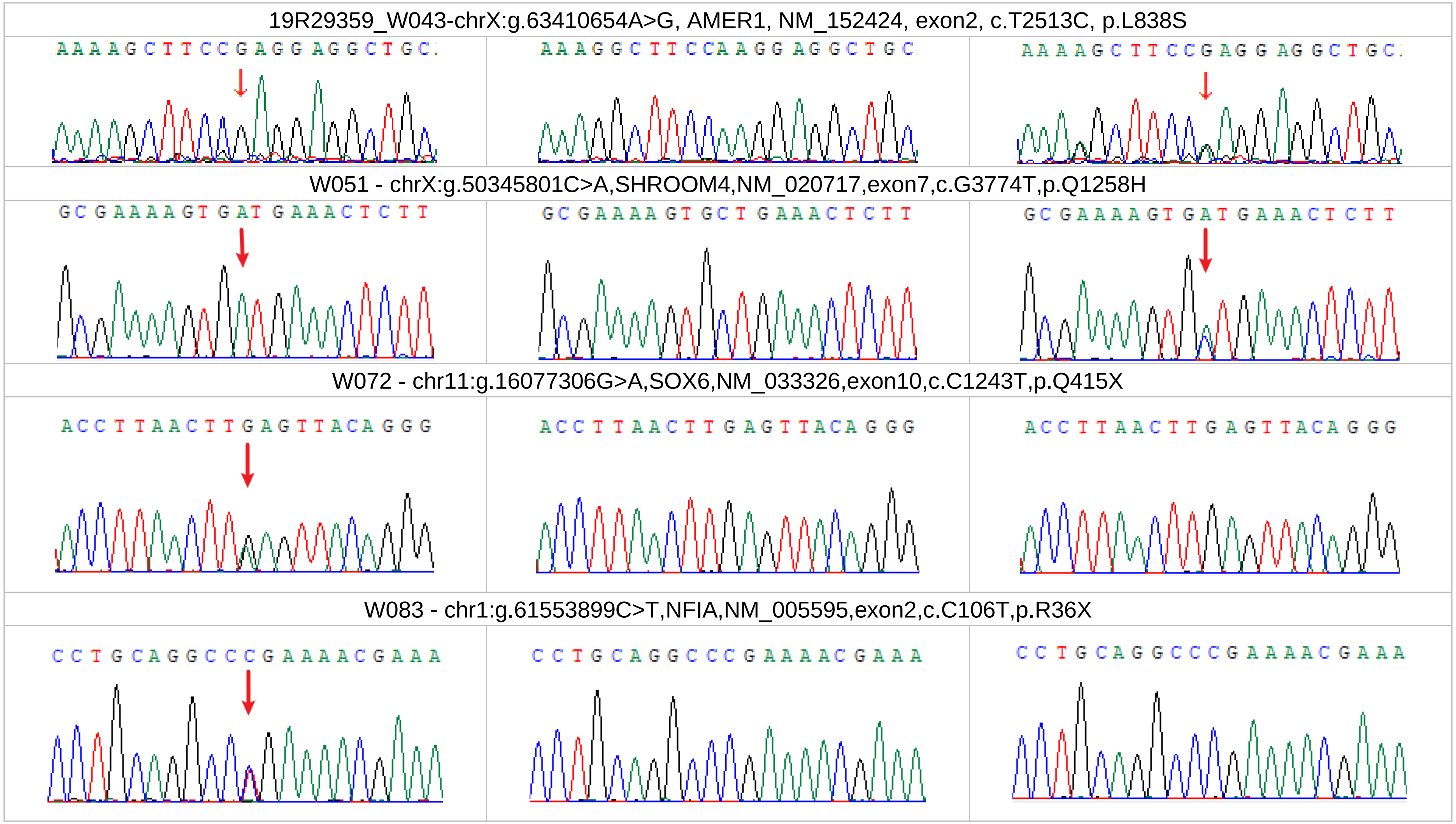
19R29359_W043-chrX:g.63410654A>G, AMER1, NM_152424, exon2, c.T2513C, p.L838S
W051 - chrX:g.50345801C>A,SHROOM4,NM_020717,exon7,c.G3774T,p.Q1258H
W072 - chr11:g.16077306G>A,SOX6,NM_033326,exon10,c.C1243T,p.Q415X
W083 - chr1:g.61553899C>T,NFIA,NM_005595,exon2,c.C106T,p.R36X

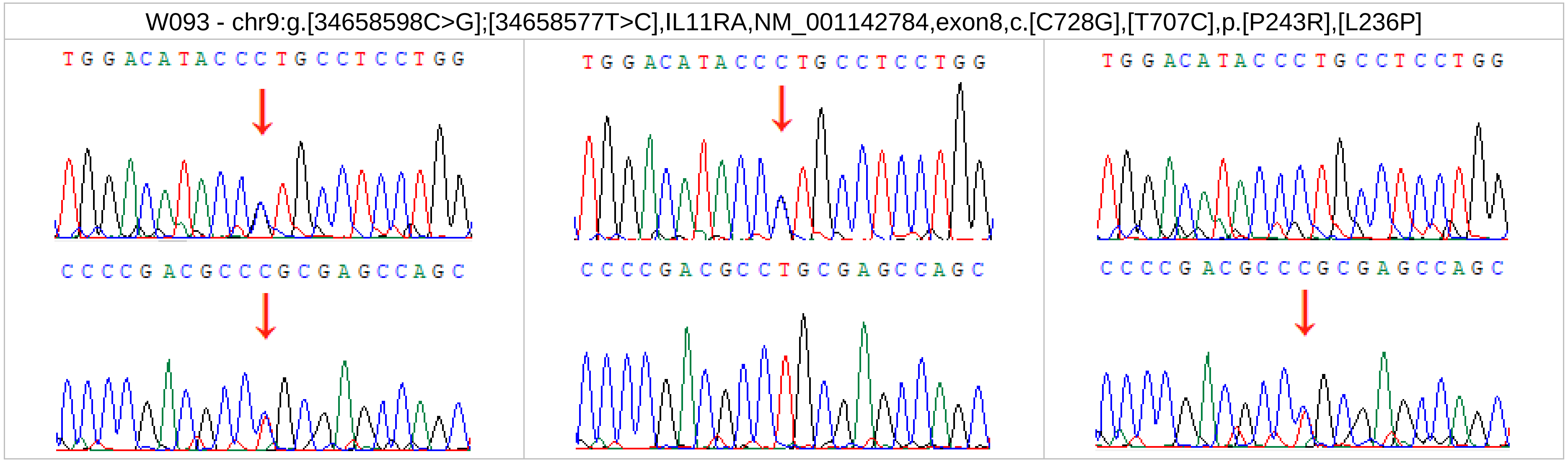

Supplementary Figure 3. Confirmation of variants by Sanger sequencing.

The screenshots of pathogenic/likely pathogenic/potentially pathogenic and candidate variants by Sanger sequencing are showed. The columns ordered by the proband, the father and the mother in a family. To those proband-only cases, the columns of parents were filled with “none”. The description of variants is above the corresponding row. To compound heterozygote, the up-down pictures correspond to the before- after of variants description. The alternative position was pointed by red arrow.

W001-chr5:g.140966698A>G, DIAPH1, NM_005219, exon3, c.T211C, p.S71P

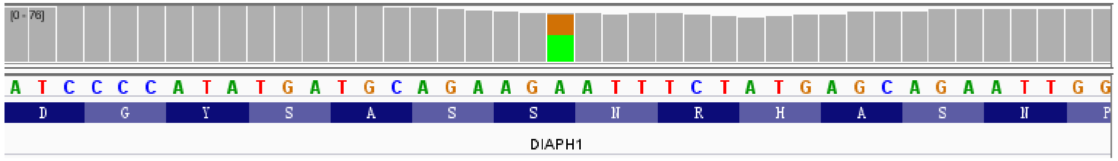

W008-chrX:g.79988961C>G, BRWD3, NM_153252, exon12, c.G1121C, p.G374A

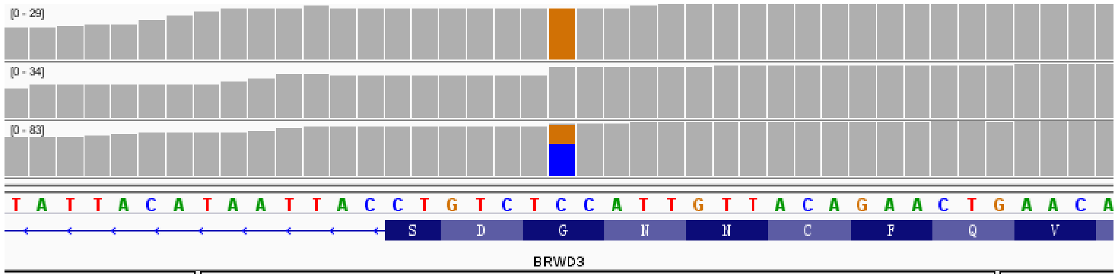

W015-chr4:g.5798945C>A, EVC, NM_153717, exon14, c.C2083A, p.L695M

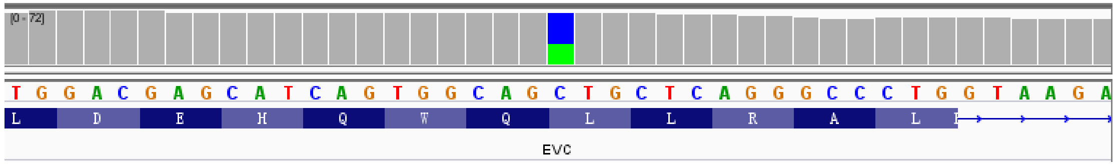

W021-chr9:g.109688611C>G, ZNF462, NM_021224, exon3, c.C2418G, p.N806K

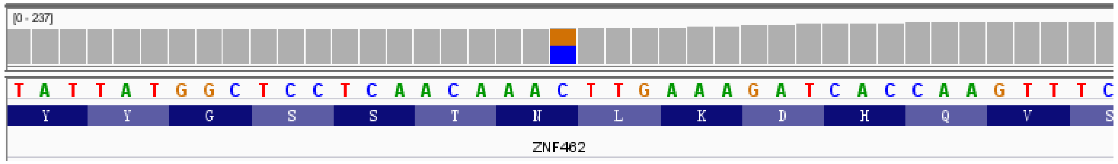

W023-chr17:g.41833050G>A, SOST, NM_025237, exon2, c.C302T, p.T101I

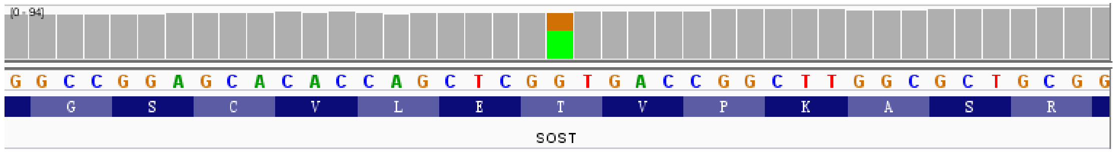

W038-chr4:g.5733339C>T, EVC, NM_153717, exon4, c.C572T, p.T191I

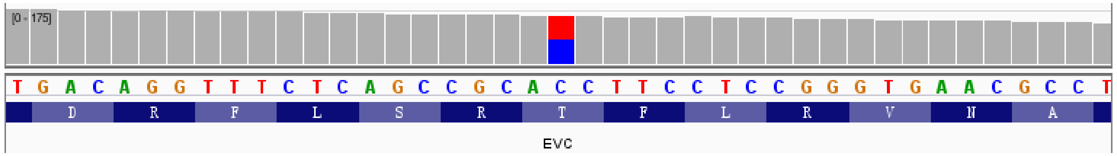

W071-chrX:g.148037734C>T, AFF2, NM_002025, exon11, c.C2159T, p.S720F

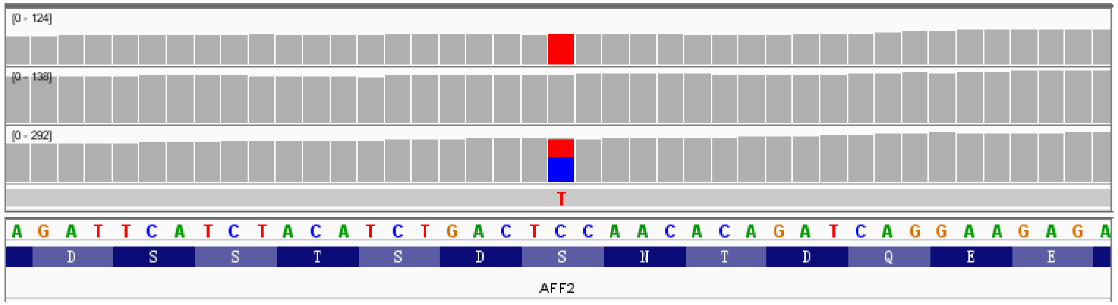

W085-chrX:g.129149626G>A, BCORL1, NM_021946, exon3, c.G2878A, p.D960N
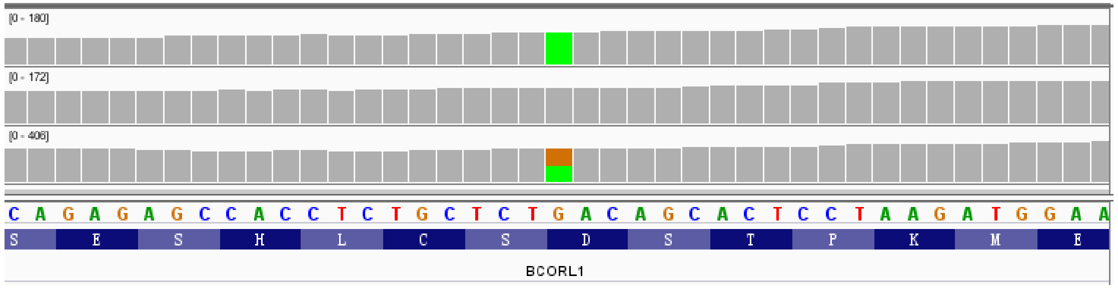
W095-chr7:g.[21726797A>C];[21639655T>C], DNAH11, NM_001277115, exon33;exon15, c.[A5702C];[T2918C], p.[E1901A];[V973A]
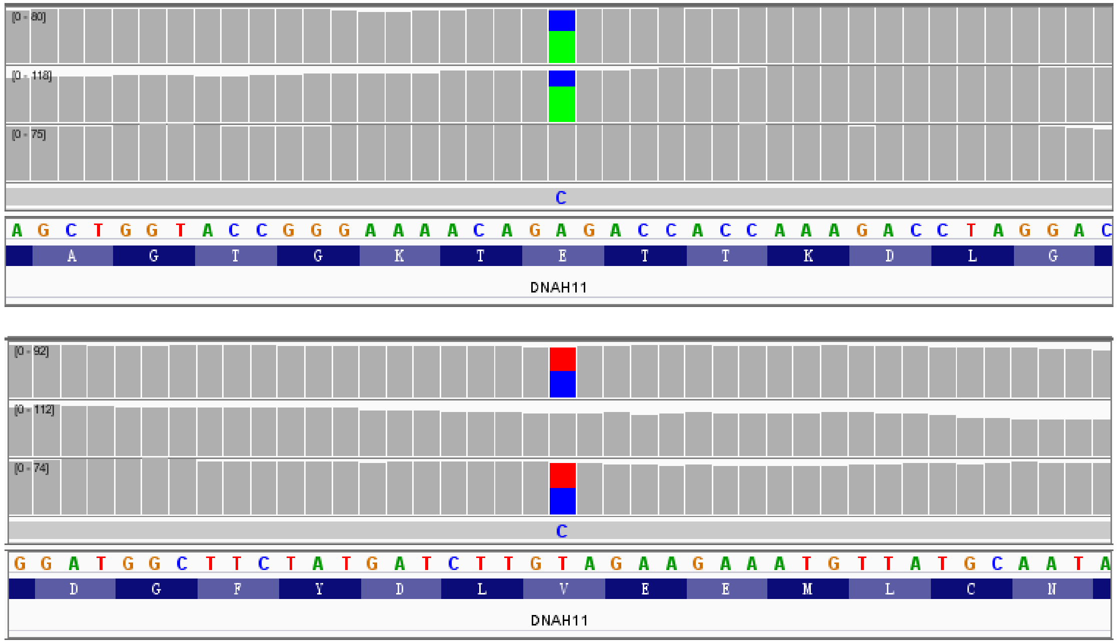

Supplementary Figure 4. Confirmation of variants by the Integrative Genomics Viewer (IGV).

The screenshots of candidate variants by IGV are showed. The rows of depth in one screenshot were ordered by the proband, the father and the mother in a family. To those proband-only cases, there is only one row of depth in the screenshot. The description of variants is above the corresponding screenshot. To compound heterozygote, the up-down screenshots correspond to the before- after of variants description. The alternative position was in the middle.

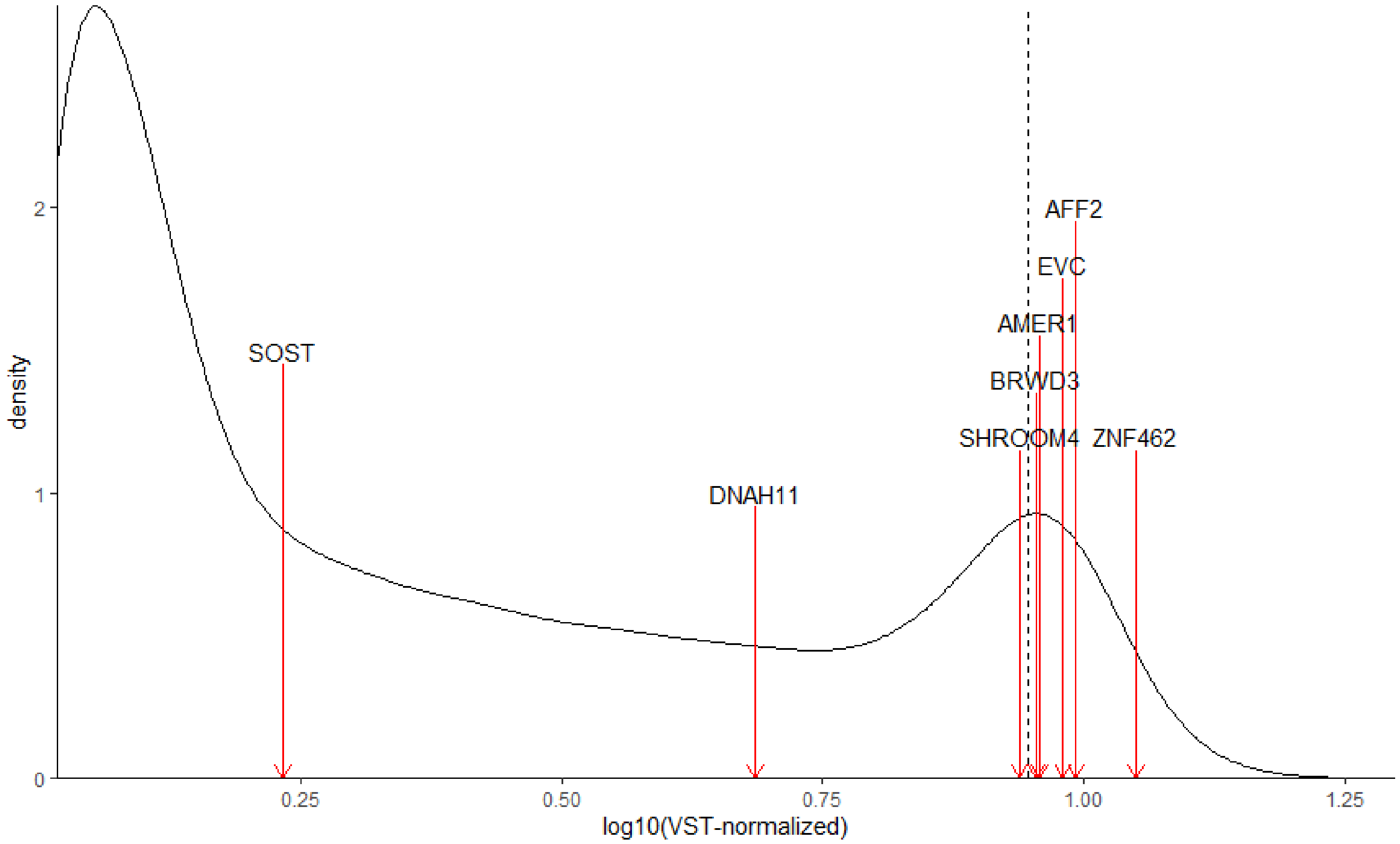

Supplementary Figure S5: Expression quantity of genes in the human cranial neural crest cells (CNCCs).
Red line with arrow: expression level of corresponding gene; black dashed line: 90% quantile of overall gene expressions.
